# Novel evidence of age-related cortical and subcortical constraints in cross-education

**DOI:** 10.1007/s11357-025-01886-1

**Published:** 2025-10-25

**Authors:** Ummatul Siddique, Ashlyn K. Frazer, Jamie Tallent, Oliver Hayman, Justin Andrushko, Matt S. Stock, Joshua Carr, Yonas Akalu, Mohamad Rostami, Sergio Uribe, Simon Walker, Dawson J. Kidgell

**Affiliations:** 1 Monash Exercise Neuroplasticity Research Unit, School of Primary and Allied Care, Monash University, Frankston, Australia; 2 School of Sport, Rehabilitation and Exercise Sciences, University of Essex, Colchester, UK; 3 School of Cardiovascular and Metabolic Health, College of Medical, Veterinary and Life Sciences, University of Glasgow, Glasgow, UK; 4 Department of Sport, Exercise and Rehabilitation, Faculty of Health and Life Sciences, Northumbria University, Newcastle-Upon-Tyne, UK; 5 School of Kinesiology and Rehabilitation Sciences, University of Central Florida, Orlando, FL USA; 6 Department of Kinesiology, Kansas State University, Manhattan, KS USA; 7 Department of Human Physiology, School of Medicine, University of Gondar, Gondar, Ethiopia; 8 Department of Medical Imaging and Radiation Sciences, School of Primary and Allied Care, Monash University, Clayton, Australia; 9 NeuroMuscular Research Center, Faculty of Sport and Health Sciences, University of Jyväskylä, Jyväskylä, Finland

**Keywords:** Cross-education, Neuroplasticity, Aging, Intracortical inhibition, StartReact, Corticospinal excitability, Strength training

## Abstract

Cross-education (CE), the phenomenon whereby unilateral strength training induces neuromuscular adaptations in the contralateral limb, is underpinned by distributed cortical and subcortical plasticity. This study evaluated acute neural responses to a single bout of metronome-paced unilateral strength training in younger (n = 17; 27 ± 6 years) and older adults (n = 18; 67 ± 5 years). Neurophysiological assessments included corticospinal excitability and inhibition via transcranial magnetic stimulation (TMS), intracortical inhibition through short-interval intracortical inhibition (SICI), and reticulospinal drive using the StartReact paradigm. Following training, younger adults exhibited a significant reduction in the responsiveness of intracortical inhibitory interneurons, reflected as a release of SICI (pre: 58.05, 95% CI 48.83–67.27; post: 68.88, 95% CI 59.69–78.06; p < 0.01), consistent with diminished GABA-_*A*_ -mediated synaptic efficacy. In contrast, older adults showed no changes (pre: 65.73, 98% CI 57.00–74.46; post: 60.71, 95% CI 52.03–69.39; p = 0.13), indicating no significant modulation of GABA-_*A*_-mediated inhibitory circuits with ageing. No changes in corticospinal excitability were observed in either group. StartReact responses remained stable across timepoints, implying insufficient acute engagement of the reticulospinal tract. Notably, younger adults exhibited a decline in rate of force development (RFD; pre: 912.70, 95% CI 821.97–1003.44 N/s; post: 791.02, 95% CI 700.29–881.75 N/s; p < 0.05), potentially reflecting central fatigue, while older adults displayed no significant change. These findings reveal age-dependent asymmetries in the neural mechanisms underpinning CE, with older adults exhibiting a lack of adaptive modulation in intracortical and subcortical systems. The absence of reticulospinal adaptation underscores the need for sustained or higher-intensity training stimuli to elicit subcortical plasticity. These results inform the development of targeted, age-sensitive neuromuscular rehabilitation strategies.

**Graphical Abstract:**

**Figure:**
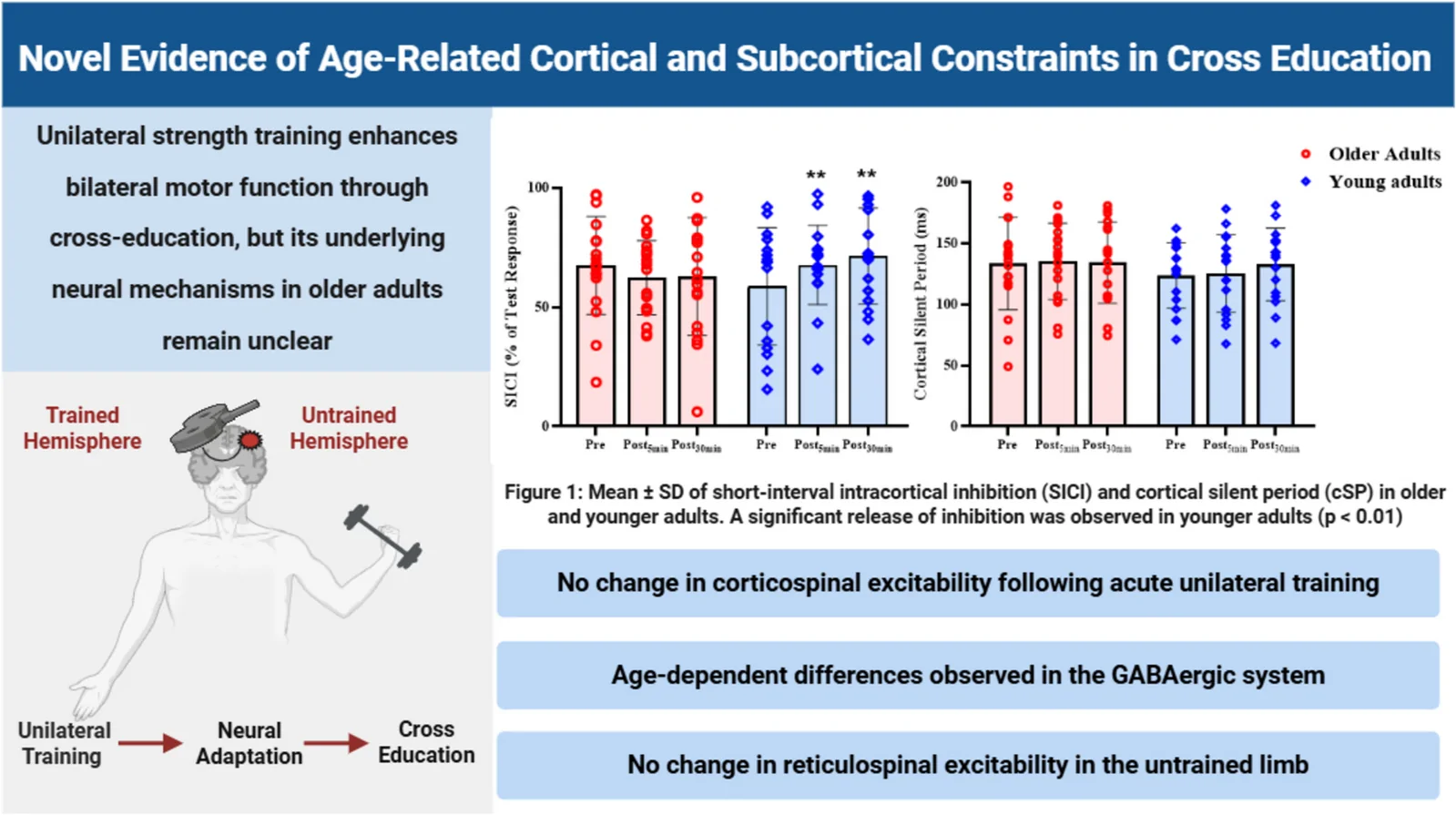

**Supplementary Information:**

The online version contains supplementary material available at https://doi.org/10.1007/s11357-025-01886-1.

## Introduction

Unilateral strength training induces bilateral motor improvements through the cross-education (CE) phenomenon [104]. This effect is primarily attributed to neural adaptations [17, 76, 98, 99], with two primary hypotheses proposed to explain this effect [45]. The first, the cross-activation hypothesis, elicit that unilateral muscle contractions posits neuroplastic changes in both motor cortices, leading to enhanced motor output bilaterally [76, 98]. The second, the bilateral access hypothesis, suggests that interhemispheric communication between the trained and untrained limb facilitates neural adaptations across the sensorimotor network [65]. Understanding hemispheric lateralisation is fundamental in mediating CE, as motor control involves differential contributions from the left and right hemispheres [57]. The extent to which CE occurs may depend on interhemispheric communication and the lateralised nature of motor plasticity. While strength gains in the untrained limb typically require multiple training sessions [78], neurophysiological changes in the untrained hemisphere can occur after just a single session [18] suggesting short-term potentiation (STP) mechanisms that contribute to long-term training effects [78].

Evidence from acute strength training studies using transcranial magnetic stimulation (TMS) has demonstrated neural adaptations in the untrained ipsilateral primary motor cortex (iM1), including increased corticospinal excitability [46, 67, 80, 86, 92], reduced intracortical inhibition [80], and decreased interhemispheric inhibition [65, 67, 92]. These adaptations reflect rapid cortical reorganization that supports cross-limb functional gains. While these mechanisms are well-established in younger adults, their presence, magnitude, and functional relevance in ageing populations remain poorly understood [12], likely due to age-related declines in synaptic plasticity and structural connectivity. Importantly, this study is the first to interrogate both cortical and subcortical motor pathway plasticity in older adults using a cross-education model. By integrating TMS-derived measures with reticulospinal assessments via the StartReact paradigm, it provides a comprehensive neurophysiological framework to evaluate how ageing modulates the responsiveness of key descending motor circuits to unilateral training. This represents a significant step toward age-informed neurorehabilitation strategies grounded in mechanistic evidence.

Unilateral injuries, often resulting from falls, become increasingly debilitating with age, with older adults (65 + years) experiencing a 30–40% annual fall risk [30, 113]. Musculoskeletal injuries can lead to prolonged immobilisation and frailty, yet CE may serve as a potential strategy to mitigate these adverse outcomes. Studies on ballistic motor training in older adults have reported increased corticospinal excitability [37, 64, 96] and either reduced [63, 64] or unchanged [62] short-interval intracortical inhibition (SICI). While CE capacity is preserved in older adults [6, 40], the magnitude of benefits appears reduced compared to younger adults [5, 91], likely due to a rostral-caudal gradient in age-related white matter atrophy within the corpus callosum, where rostral centres exhibit a greater degree of age-related atrophy [66]. Although a meta-analysis found no significant age-related differences in corticospinal excitability following ballistic training, younger adults exhibited greater CE effects [118], suggesting that age-related declines in interhemispheric connectivity may reduce neural efficiency to mediate CE [44, 102, 106, 116].

Despite a substantial body of research examining the neural substrates of CE following unilateral strength training [71, 82], findings remain equivocal. While some studies report no measurable changes in ipsilateral corticospinal excitability despite observable performance gains in the untrained limb [99], others demonstrate CE in the absence of detectable corticospinal modulation, implicating the recruitment of non-corticospinal pathways [24]. These inconsistencies underscore a significant gap in the current understanding of CE, i.e., the overreliance on corticospinal metrics may obscure contributions from alternative descending systems. Given the bilateral influence and structural resilience of pathways such as the cortico-reticulospinal tract, especially in the context of age-related corticospinal decline, there is a pressing need to systematically investigate the role of these subcortical circuits in mediating strength transfer. Advancing our understanding of these mechanisms may reveal underutilized targets for neuromotor rehabilitation across the lifespan.

The reticulospinal tract is a promising candidate for force production due to its bilateral motor influence [31, 52]. Originating in the brainstem, the reticulospinal tract plays a vital role in motor control via polysynaptic connections with spinal motoneurons, facilitating coordinated activation of large muscle groups [32, 68, 94]. Its role in motor recovery is well documented in stroke and spinal cord injury populations [3, 28, 103, 110], yet its involvement in CE remains largely unexplored. Evidence from non-human primates suggests that strength training enhances reticulospinal tract excitability without affecting corticospinal tract excitability [53], indicating that the reticulospinal tract may be a primary driver of strength-related neural adaptations. Given its bilateral influence, the reticulospinal tract is a strong candidate for undergoing neural adaptation following unilateral strength training, particularly in ageing populations where corticospinal function has been reported to decline [10, 22]. Despite its potential role, reticulospinal tract adaptations in older adults remain poorly understood, partly due to challenges in non-invasively assessing reticulospinal tract activity. However, the StartReact paradigm, which utilises startling auditory stimuli to activate the reticular formation [33, 58], offers an indirect method for assessing reticulospinal tract function. StartReact has been shown to accelerate reaction times and enhance the maximal motor unit discharge rate and rate of force development (RFD; [2, 41, 83, 110]), making it a valuable tool for investigating reticulospinal contributions to CE.

Beyond the reticulospinal tract, the corticoreticular pathway has also been proposed as a key mediator of CE [12]. Given its bilateral projections, the corticoreticular pathway has been proposed as a candidate mechanism supporting CE, particularly in ageing populations where corticospinal plasticity may be reduced [12, 101]. While direct evidence is lacking, non-invasive techniques such as TMS may allow for partial activation of subcortical circuits, and latency-based motor evoked potential (MEP) analyses could offer indirect insights into the respective contributions of corticospinal and reticulospinal pathways [42, 100].

The primary purpose of this study was to investigate how ageing influences acute neuroplasticity (STP) in descending motor pathways following unilateral strength training. Specifically, we aimed to examine the immediate neural adaptations within corticospinal, corticoreticular, and reticulospinal circuits in response to a single session of metronome-paced unilateral training in both younger and older adults. By focusing on the untrained, contralateral limb, we sought to delineate the neurophysiological mechanisms underlying CE and determine whether ageing alters the engagement of cortical and subcortical motor pathways. We hypothesised that younger adults would exhibit greater modulation of corticospinal and intracortical excitability, whereas older adults would show blunted responses, potentially reflecting reduced plasticity in these circuits. Furthermore, we posited that subcortical pathways, particularly the reticulospinal tract, may be less responsive to acute training stimuli in older adults, contributing to age-related constraints in CE. This investigation provides a mechanistic foundation for developing age-specific, pathway-targeted rehabilitation strategies aimed at preserving or restoring unilateral motor function.

## Methods

### Participants

A total of 35 participants (18 older adults: 6 males, 12 females; mean age: 67 ± 5 years, height: 167 ± 9 cm, body mass: 75 ± 14 kg, BMI: 27 ± 6 kg/m^2^; 17 young adults: 10 males, 7 females; mean age: 27 ± 6 years, height: 170 ± 10 cm, body mass: 69 ± 12 kg, BMI: 24 ± 3 kg/m^2^) were recruited through community advertisements. Participants were screened for musculoskeletal or neurological injuries using the TMS Safety Questionnaire [69], and no hearing impairments were reported. All participants self-reported as sedentary and had not engaged in strength training in the previous 12 months. Hand dominance was assessed using the Edinburgh Handedness Inventory [89]. The study was approved by the Monash University Human Research Ethics Committee (Project ID: 30882) and conducted in accordance with the Declaration of Helsinki. Written informed consent was obtained from all participants.

### Experimental design & strength exercise protocol

**Fig. 1:**
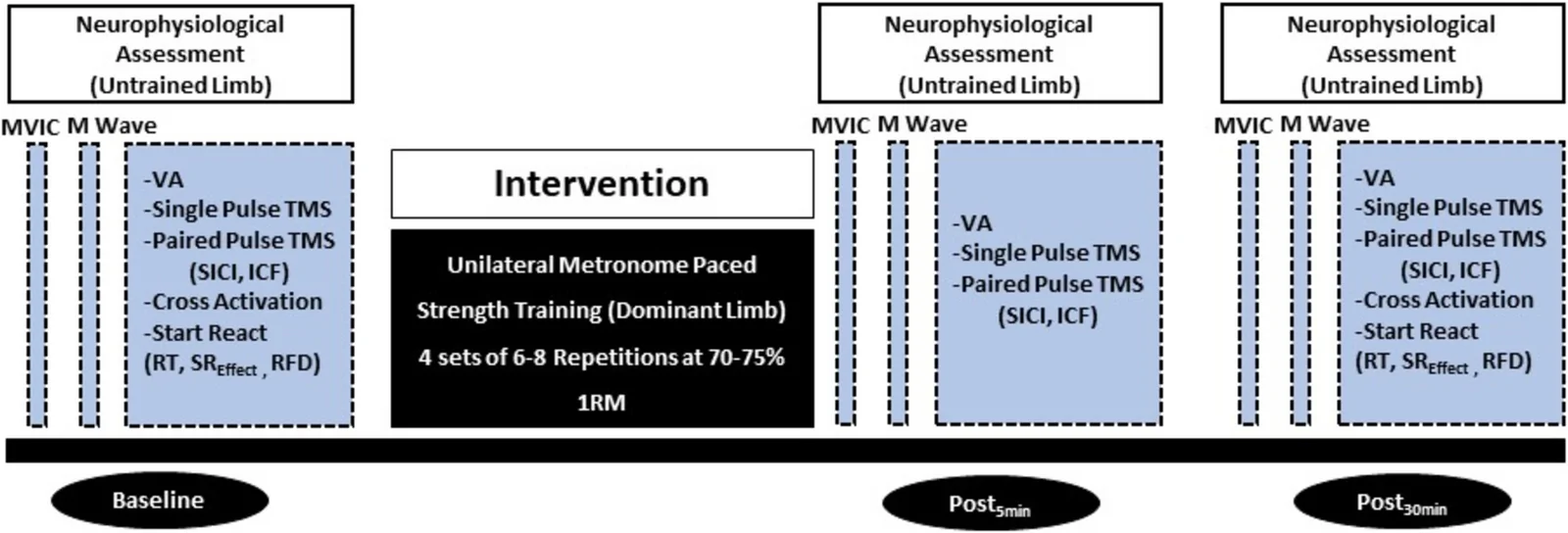
Schematic representation of the experimental design with measures obtained before and following a single session of unilateral metronome-paced strength training (MPST). 1RM: one repetition maximum; MVF: Maximum Voluntary Force; M-WAVE: Maximum compound potential; VA: Voluntary Activation; TMS: Transcranial Magnetic Stimulation; SICI: Short interval intracortical inhibition; ICF: Intracortical facilitation; RT: Reaction Time; SR_Effect_: StartReact Effect; RFD: Rate of Force Development

Participants attended the laboratory on two separate occasions, spaced one week apart. The initial visit served as a familiarisation session, during which participants' anthropometric data (height and weight) were recorded. During this session, participants were introduced to the experimental procedures, including TMS and other assessment protocols. They also performed practice trials of maximum voluntary force (MVF) measurements and the StartReact protocol to ensure adequate familiarity with the procedures before the primary testing session. The second visit constituted the main testing session, during which various physiological outcome measures, including MVF, TMS, peripheral electrical stimulation, and the StartReact protocol, were evaluated. These measures were assessed in the untrained limb both before and after a single session of strength exercise. The testing battery was administered twice: once immediately after strength training (~ 5 min post-exercise) and again during the acute recovery phase (~ 30 min post-exercise), as illustrated in Fig. 1, which outlines the study design.

Dynamic strength of the elbow flexors was assessed in the trained (dominant) and untrained (non-dominant) arms of both young and older participants using the one-repetition maximum (1-RM) test. Participants initially estimated their 1-RM and then performed a standard biceps curl with a dumbbell while standing against a wall to maintain proper form and minimize compensatory movements. A three-minute rest was provided between trials to reduce fatigue. Weight was increased in 0.5 kg increments until failure, with the heaviest successful lift recorded as the 1-RM. Most participants reached their 1-RM within three to five trials. The 1-RM for the trained arm was first determined to establish the training load at 70–75% of the participant's 1-RM [79], and the same procedure was repeated for the untrained arm.

Participants then completed a single session of unilateral strength training, consisting of four sets of 6–8 biceps curls at 70–75% 1-RM. A metronome regulated the tempo: three seconds for the concentric phase and four seconds for the eccentric phase [72]. A two-minute rest was provided between sets to minimize fatigue and maintain performance. Surface electromyography (sEMG) data were recorded from both arms throughout the session to assess muscle activation and neuromuscular activity.

### Surface electromyography (sEMG) and maximum voluntary force (MVF)

sEMG activity of the untrained biceps brachii was recorded using bipolar Ag–AgCl electrodes, following SENIAM guidelines [61]. The electrode area was prepared by abrasion and cleansing with 70% isopropyl alcohol [51]. Electrodes were placed 2 cm apart over the muscle belly, one-third of the distance from the cubital fossa [109]. A grounding strap was secured around the wrist to maintain signal quality. sEMG signals were amplified (× 1000), bandpass filtered (13–1000 Hz), and digitized at 2 kHz. Data were recorded and analysed using the PowerLab 4/26 system (AD Instruments, Bella Vista, Australia).

For MVF assessment, participants were instructed to perform a maximal voluntary isometric contraction of the elbow flexors while seated with their elbows flexed at 90°, maintaining a supinated hand position. A force transducer (Futek Force Transducer LSB302, Melbourne) was positioned at the forearm level, aligned with the wrist and participants exerted maximum force against it for 3 s. Three trials were completed, with a 3-min rest between trials to minimise fatigue [109]. Consistency was ensured by allowing no more than 5% variation in force across trials. The highest value was recorded as the participant's MVF [72]. Force was measured at baseline, 5, and 30 min post-exercise to assess changes in isometric force over time.

### Transcranial magnetic stimulation (TMS) and cross activation

TMS was applied over the iM1 using a BiStim unit connected to two Magstim 200^2^ stimulators (Magstim Co, Dyfed, United Kingdom) to elicit MEPs in the untrained (non-dominant) biceps brachii. A 90 mm circular coil was positioned at a 45° angle to direct a posterior-to-anterior current flow within the iM1. The motor hotspot for the non-dominant biceps brachii was located, and the active motor threshold (AMT) was determined as the lowest intensity that elicited MEPs exceeding 200 µV in at least five of 10 pulses, while contracting to 10% of the participant’s MVF [72]. AMT was reassessed and adjusted if necessary after the strength exercise session.

Corticospinal excitability was assessed via MEP amplitude, and corticospinal inhibition through the cortical silent period (cSP), reflecting GABA_B_-mediated inhibition. Five single-pulse TMS pulses were delivered at 130%, 150%, and 170% of AMT while participants maintained a 10% MVF isometric contraction [97], with pulses spaced 10 s apart and synchronized to a metronome [72].

Corticocortical excitability and inhibition were assessed using paired-pulse TMS, with five trials recorded per condition. Intracortical facilitation (ICF) was used to assess glutamatergic excitatory activity, whereas short-interval intracortical inhibition (SICI) reflected GABA_A_-mediated inhibitory processes. Test stimuli were delivered at 130% of AMT and conditioning stimuli at 80%, with interstimulus intervals of 3 ms for SICI and 10 ms for ICF [84].

TMS assessments were performed at baseline, 5, and 30 min post-exercise. Electromyography root mean square (EMGrms) was recorded during the 100 ms interval before each TMS pulse to account for fluctuations. All data were captured and analysed using LabChart™ software (version 8.1.24, ADInstruments, Bella Vista, Australia).

Cross-activation was assessed by delivering 10 single-pulse TMS stimulations to the iM1 (130% AMT) during a maximal voluntary contraction of the dominant biceps, with EMG recorded from the non-dominant (resting) biceps [46]. Data was recorded at baseline and 30 min post-exercise to assess training-induced changes in ipsilateral corticospinal excitability.

### Maximum compound action potential and central activation ratio (CAR)

Peripheral muscle responses in the untrained (non-dominant) biceps brachii were assessed via supramaximal stimulation of the brachial plexus at Erb’s point (DS7A; Digitimer, UK). Stimulation was applied using 3.2 cm round electrodes, with the cathode positioned over the supraclavicular fossa and the anode on the acromion. Starting at 20 mA, intensity was gradually increased in 10 mA increments until the evoked sEMG response M-wave plateaued. To ensure maximal activation, an additional 20% above the plateau current was applied [90]. Three single pulses were delivered at this intensity (5–8 s apart), and the largest peak-to-peak M-wave amplitude was recorded as the maximal M-wave (M_MAX_) [1]. Measurements were taken at baseline, and 5- and 30-min post-exercise track changes in peripheral muscle activity over time.

Voluntary activation was assessed using the central activation ratio (CAR), determined after the participant’s MVF test. While maintaining a near-maximal contraction (> 90% MVF), a supramaximal stimulus (M_MAX_) was delivered to Erb’s point (200 µs pulse width) to evoke a superimposed twitch [49]. This was then followed by stimulation at rest to evoke a resting twitch [109]. CAR was measured at baseline, and at 5- and 30-min post-exercise to monitor changes in voluntary drive.

### StartReact protocol

The StartReact protocol [112] was used to assess reticulospinal tract activity in the untrained limb. Participants sat comfortably with relaxed shoulders, elbows flexed at 90°, and hands in a supinated position. A force transducer was placed at the forearm level to measure force output. In response to an LED light positioned one meter in front of them at eye level, participants were instructed to rapidly flex their elbow against the transducer. Prior to testing, practice trials involving rapid contractions and exposure to auditory stimuli ensured participants were familiar with the procedure.

The protocol included three randomized conditions: (1) LED light alone for visual reaction time (VRT), (2) LED light with a soft sound (80 dB, 500 Hz, 50 ms) for visual-auditory reaction time (VART), and (3) LED light with a loud sound (115–120 dB, 500 Hz, 50 ms) for visual startle reaction time (VSRT). Auditory stimuli were delivered via a speaker placed one meter from the participant. Thirty trials were completed with 3–6 s intervals to minimise predictability and reduce habituation. To ensure reliability of this protocol, pilot testing yielded intraclass correlation coefficient (ICC) values of 0.72 for VRT, 0.75 for VART, and 0.82 for VSRT. Our data aligns with the findings from Colomer Poveda et al. (2023) [26] further validating our protocol.

### Data analysis

TMS data were analysed to assess EMGrms activity in the untrained biceps brachii during the 100 ms prior to each stimulus, at baseline and 5-and 30-min post-exercise. Trials with pre-stimulus EMGrms exceeding 5 ± 1% of maximal EMGrms were excluded. MEP peak-to-peak amplitudes were extracted using LabChart software (ADInstruments, Australia), normalized to M_MAX_, and multiplied by 100 to account for peripheral muscle changes. The silent period duration was assessed using single-pulse TMS at 130–170% of AMT, determined through visual inspection with onset marked from stimulus delivery and cessation defined by the return of sEMG activity to pre-stimulus levels.

To differentiate neural inputs, early- and late-phase MEPs were analysed, distinguishing monosynaptic corticospinal contributions from poly- or oligosynaptic corticoreticular and other corticofugal pathways. The mean MEP amplitude (mV) in the untrained biceps brachii was evaluated across stimulation intensities ranging from 100 to 170% of AMT. Two specific time windows were analysed from MEP onset: **the early phase (0–8 ms)**, reflecting corticospinal input, and the **late phase (8–24 ms)**, representing corticoreticular or other descending contributions. These time frames were selected based on reticular formation recordings by Fisher et al. (2012) [42], which identified late neural activity persisting up to 25 ms post-stimulation. All data were processed and analysed using a custom macro in LabChart.

SICI and ICF ratios were calculated to analyse paired-pulse data across different time points. These ratios were expressed as a percentage of unconditioned single-pulse MEP amplitudes, recorded at 130% of AMT. To assess interhemispheric asymmetries in corticospinal excitability and inhibition, we calculated the laterality index (LI) using the mean difference in excitability and cortical silent period duration between the two hemispheres. Following the approach outlined by Cramer et al. (1997) and Langan et al. (2010) [29, 75], the LI was computed for each condition using the formula:

$$Laterality Index\left(LI\right)=\frac{(L-R)}{(L+R)}$$

where **L** represents the left hemisphere and **R** represents the right hemisphere.

A score of **1** indicates complete lateralization to the left hemisphere, while a score of **−1** reflects complete lateralization to the right hemisphere. Additionally, any value close to zero would indicate bilateral activation. In this study, a positive LI score suggests greater excitability in the dominant M1 (left hemisphere, right arm), whereas a negative score indicates greater excitability in the non-dominant hemisphere (right hemisphere, left arm).

Additionally, central activation ratio (CAR) was computed to evaluate overall neural drive to the elbow flexors. CAR was determined using the following formula:

$$CAR \left(\%\right)=\left(\frac{Maximal Force}{Maximal Force+ITT}\right) \times 100$$

Here, maximal force represents the MVF achieved during maximal isometric testing, while maximal force plus ITT (Interpolated Twitch Technique) represents the MVF achieved via supramaximal electric stimulation [73].

For reticulospinal tract activity, reaction times were assessed under three conditions (VRT, VART, and VSRT). Reaction time was defined as the onset latency of sEMG activity in the untrained biceps brachii, with trials exceeding ± 3 standard deviations (SD) above baseline discarded. To ensure accuracy, each trial was manually inspected for errors in sEMG onset detection, as data were analysed using a custom-written macro in LabChart. Additionally, the StartReact Effect (SREffect), calculated as the difference between VART and VSRT, was used to assess reticulospinal tract drive [50]. A larger SREffect indicated greater reticulospinal tract excitability from the loud sound compared to corticospinal excitability from the soft sound [114].

$${SR}_{Effect}=(VART-VSRT)$$

The rate of force development (RFD) was measured over two intervals (0–50 ms and 50–100 ms) following force initiation, defined as the point at which the force signal exceeded three standard deviations above baseline [1, 26]. EMGrms amplitude was also analysed within these time windows after EMG onset.

### Power and statistical analysis

Power analysis was conducted using G*Power software, with corticospinal excitability as the primary outcome due to the lack of research on reticulospinal activity post-strength training in both older and young adults. Based on a previous study [109] reporting a within-group effect size (Cohen’s f = 0.44) for corticospinal excitability changes in young adults, we set power at 0.80 and α at 0.05. A repeated-measures ANOVA indicated that 10 participants per group were needed. To account for potential attrition (20%), we recruited 18 older adults and 17 young adults.

Statistical analyses were performed using SPSS software (IBM SPSS Statistics, version 26) and graphical representations were created in GraphPad Prism. Normality of key outcome variables (MEP, cSP, SICI, ICF, cross activation, LI, CAR, reaction time, SREffect, RFD, and EMGrms) was assessed with the Shapiro–Wilk test. For non-normally distributed variables, we examined residual distributions through histogram visualisation and confirmed homoscedasticity to ensure the validity of our analyses [48].

To examine the effects of a single strength exercise session on cortical, corticospinal, corticoreticular, and reticulospinal responses, we employed a Linear Mixed Model with Repeated Measures (LMM_RM_) (Wilkinson et al., 2023) using Maximum Likelihood (ML) parameter estimation. This approach accounted for both fixed and random effects, providing a robust framework for analysing longitudinal and complex datasets. The primary outcome measures included corticospinal excitability and inhibition (MEPs and cSP at 130%, 150%, and 170% of AMT), SICI, ICF, corticoreticular responses (early and late MEPs at 100%, 130%, 150%, and 170% of AMT), LI, cross activation, CAR, reaction time, reticulospinal tract drive, RFD, and EMGrms.

The model incorporated main effects for time (Pre, Post5, Post30), age group (older vs. young), StartReact cue (VRT, VART, VSRT), and time window (0–8 ms and 8–24 ms for early and late MEPs; 0–50 ms and 50–100 ms for RFD and EMGrms), as well as their interactions (e.g., age group × time, age group × time × StartReact cue, and age group × time × StartReact cue × time window). Participants were included as random effects. Post-hoc comparisons were conducted using the Sidak multiple comparison test. Baseline differences between groups were assessed using unpaired *t*-tests. Effect sizes were interpreted following Cohen’s d guidelines, where values of 0.2, 0.5, and 0.8 were classified as small, moderate, and large, respectively [23]. Statistical significance was set at *p* < 0.05 for all analyses. Data are presented as marginal means (EMM) with 95% CI unless otherwise stated. Raw means ± SD are also reported where applicable. Figures depict raw individual values, whereas tables report outcomes from mixed-model analyses.

## Results

### Baseline electrophysiological and strength measures

A total of 35 participants completed the study with no withdrawals or adverse events reported. Unpaired *t*-test or Mann Whitney Test was used to examine baseline difference between the two groups. Analysis revealed no significant between-group differences at baseline for 1-RM strength (*p* = 0.399), 1-RM relative strength (*p* = 0.055), 1-RM EMGrms (%M_Max_) (*p* = 0.885) MVF (*p* = 0.181), MVF EMGrms (%M_MAX_) (*p* = 0.925), cSP Duration (*p* = 0.709), SICI (*p* = 0.972), ICF (*p* = 0.633), CAR (*p* = 0.691), SREffect (*p* = 0.820). However, significant baseline differences were observed for MVF Relative Strength (*p* = 0.035, *d* = 0.75), AMT (*p* = 0.030, *d* = 0.66) and cross activation (*p* = 0.022, *d* = 0.43) (Table 1).

**Table 1:** Mean (95% CI) values for M_MAX_ amplitude, ESO M_MAX_, single pulse TMS prestimulus rmsEMG and paired pulse TMS prestimulus EMGrms, 1-RM, 1-RM EMGrms (%M_MAX_), MVF, Relative Strength and MVF EMGrms for untrained biceps brachii prior to and following a single session of unilateral strength-training

	**Older Adults**			**Young Adults**		
	**Pre**	**Post**_**5min**_	**Post**_**30min**_	**Pre**	**Post**_**5min**_	**Post**_**30min**_
**M**_**MAX**_** (mV)**	6.77 (4.97–8.57)	6.84 (5.09–8.59)	6.81 (4.91–8.71)	11.26 (8.93–13.59)	9.89 (7.63–12.15)	9.75 (7.27–12.23)
**ESO M**_**MAX**_** (mA)**	125 (111–140)			106 (87–124)		
**SPEMGrms (%M**_**MAX**_**)**	0.99 (0.77–1.22)	0.97 (0.74–1.19)	1.06 (0.84–1.28)	0.96 (0.73–1.19)	1.13 (0.89–1.36)	1.21 (0.98–1.44)
**PPEMGrms (%M**_**MAX**_**)**	1.15 (0.74–1.57)	1.26 (0.84–1.67)	1.47 (1.06–1.89)	1.03 (0.59–1.45)	1.43 (1.01–1.86)	1.28 (0.85–1.71)
**1-RM (kg)**	9 (8–11)			11 (9–13)		
**1-RM Relative Strength**	0.12 (0.11–0.14)			0.15 (0.13–0.18)		
**1-RM EMGrms (%M**_**MAX**_**)**	5.37 (3.03–7.71)			5.19 (4.41–5.98)		
**MVF (N)**	78.38 (62.09–94.66)	79.65 (63.36–95.93)	80.56 (64.27–96.84)	94.40 (77.64–111.16)	89.49 (72.74–106.25)	85.81 (69.05–102.57)
**Relative Strength (N/kg)**	**1.05**^**#**^ **(0.84–1.26)**			**1.35**^**#**^ **(1.16–1.55)**		
**MVF EMGrms (% M**_**MAX**_**)**	6.27 (4.66–7.87)	6.02 (4.32–7.73)	6.29 (4.33–8.25)	6.16 (4.35–7.97)	6.36 (4.85–7.87)	6.87 (4.95–8.79)

M_MAX_ maximum compound action potential*, ESO* electrical stimulator output, *EMG* electromyography, *mA* milliamp*s, PPEMGrms* paired-pulse EMG root-mean-squared, *SI* stimulator input, *SPEMGrms* single-pulse EMG root-mean-squared, *1-RM* one repetition maximum*, MVIC* maximum voluntary isometric contraction

^#^ denotes *p* < 0.05 baseline differences in older group compared with young adults for MVF Relative Strength

### Maximum Voluntary Force (MVF) and Prestimulus EMGrms

**Fig. 2:**
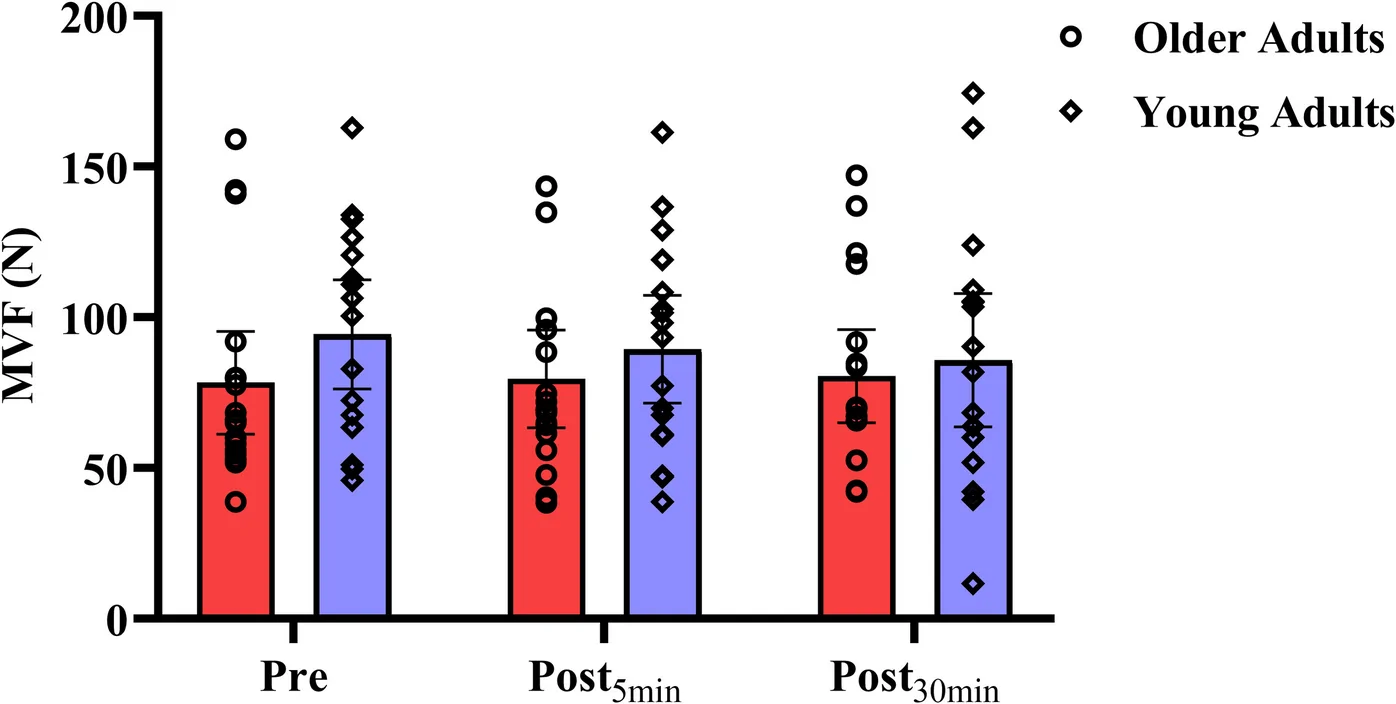
Changes in maximal voluntary force (MVF) of the untrained biceps brachii (Mean ± 95% CI) across all time points in older and younger adults

Analysis of MVF in the untrained limb following a single bout of strength exercise showed no significant main effects for time (*F*_2, 70_ = 0.592, *p* = 0.56), age group (*F*_1, 35_ = 0.879, *p* = 0.36) or time × age group interaction (*F*_2, 70_ = 1.671, *p* = 0.19) (Fig. 2).

For single-pulse pre-stimulus EMGrms, there was no significant time × age group interaction (*F*_2, 70_ = 1.020, *p* = 0.37). Likewise, the paired-pulse pre-stimulus EMGrms showed no significant time × age group interaction (*F*_2, 70_ = 0.754, *p* = 0.47).

### Corticospinal excitability (CSE) and cortical silent period (cSP)

Analysis of TMS responses revealed a significant main effect for time (*F*_2, 2065_ = 3.31, *p* = 0.037), MSO (*F*_3, 2065_ = 697.56, *p* < 0.001), and a significant MSO × age group interaction (*F*_3, 2065_ = 10.52, *p* < 0.001). No significant effects were observed for age group (*F*_1, 35_ = 0.01, *p* = 0.94), or the time × age group interaction (*F*_2, 2065_ = 1.42, *p* = 0.24).

For cSP, significant main effects were observed for time (*F*_2, 1530_ = 7.29, *p* < 0.001), MSO (*F*_2, 1530_ = 667.48, *p* < 0.001), along with a significant time × age group interaction (*F*_2, 1530_ = 6.87, *p* = 0.001) and time × age group x MSO interaction (*F*_4, 1530_ = 2.81, *p* = 0.024). No significant main effect was found for age group (*F*_1,35_ = 0.49, *p* = 0.48). Post hoc comparison in older adults showed that at 170% AMT, cSP increased significantly at Post_30min_ compared to baseline (MD = 8.96 ms, *p* = 0.005, 95% CI [2.778, 15.137], *d* = 0.22).

**Fig. 3:**
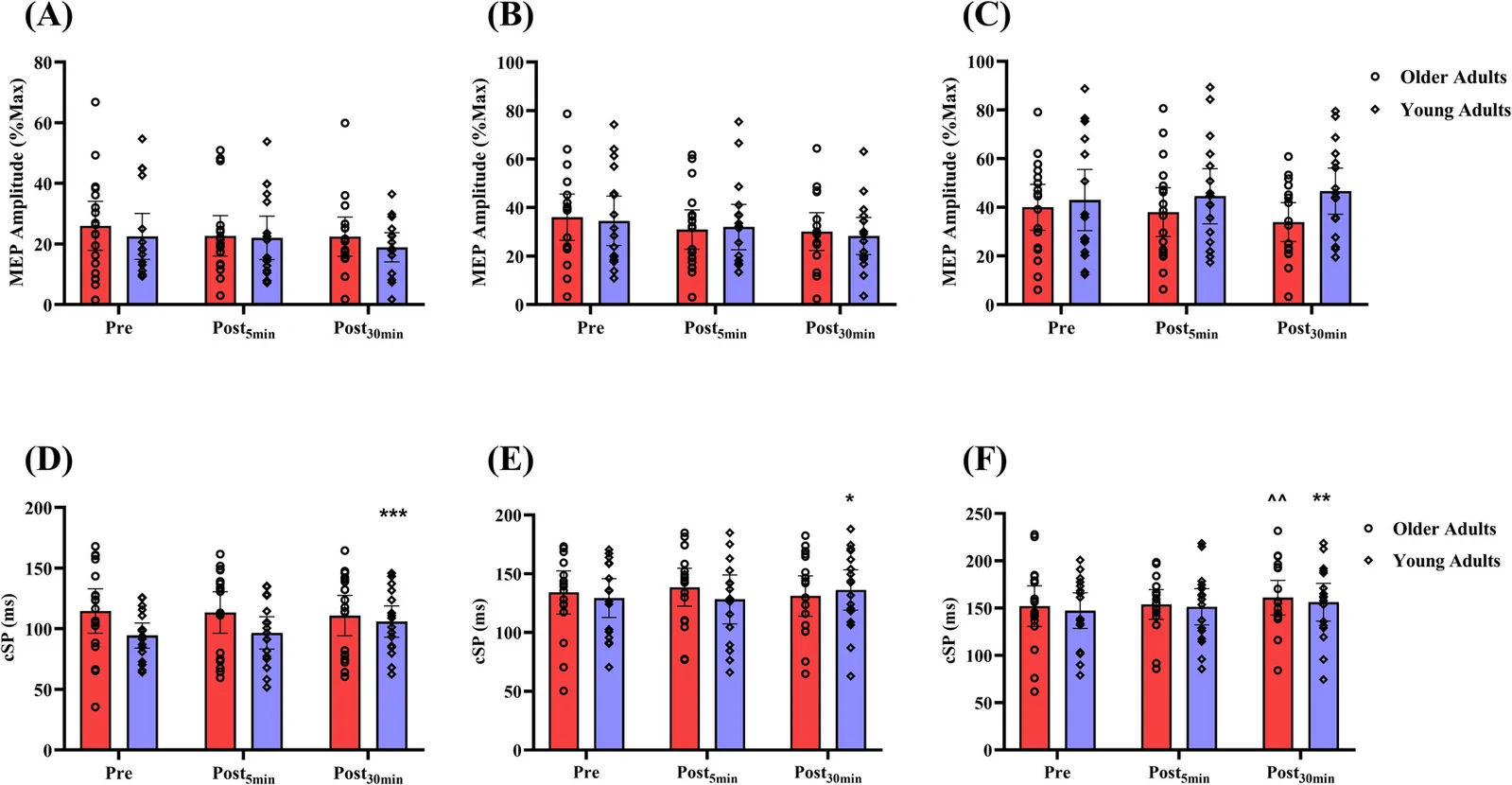
MEP amplitude at (**A**) 130% AMT (**B**) 150% AMT (**C**) 170% AMT of untrained biceps brachii, expressed as M_MAX_ (Mean ± 95%CI) and cSP duration(ms) (Mean ± 95%CI) at (**E**) 130% AMT, (**F**) 150% AMT, and (**G**) 170% AMT of the untrained biceps brachii in both older and young adults. Significant increases at 5- and 30-min post-exercise is denoted by *(p < 0.05); **(*p* < 0.01) & ***(*p* < 0.001) in young adults and ^^(*p* < 0.001) in older adults

Additionally, young adults also exhibited a consistent increase in cSP across all MSO intensities at Post_30min_ from baseline. At 130% AMT, a significant increase was observed at Post_30min_ (MD = 11.48 ms, *p* < 0.001, 95% CI [5.098, 17.854], *d* = 0.50). Similar increases were seen at 150% AMT (MD = 6.91 ms, *p* = 0.035, 95% CI [0.489, 13.331], *d* = 0.21) and 170% AMT (MD = 9.09 ms, *p* = 0.006, 95% CI [2.594, 15.564], *d* = 0.24). Additionally, no significant between group difference were observed between older and young adults across all MSO levels at all time points (*p* > 0.05) (Fig. 3 and Table 2).

**Table 2:** Mean (95% CI) for the change in iM1 MEP (%M_MAX_) at 130, 150, and 170% of AMT, SICI, and ICF for older and young adults following a session of metronome paced unilateral strength training

		**AMT**	**Corticospinal Excitability MEP(%M**_**MAX**_**)**			**Cortical Silent Period cSP (ms)**			**SICI** **(% test response)**	**ICF** **(% test response)**
			**130%AMT**	**150%AMT**	**170%AMT**	**130%AMT**	**150%AMT**	**170%AMT**		
**Older Adults**	**Pre**	**47**^**#**^ **(44–51)**	25.99 (19.73–32.25)	36.03 (29.77–42.29)	40.01 (33.75–46.27)	114.62 (99.79–129.45)	134.09 (119.26–148.92)	152.09 (137.26–166.92)	65.73 (57.00–74.46)	147.55 (125.31–169.79)
	**Post**_**5min**_	45 (42–49)	22.68 (16.42–28.94)	30.94 (24.68–37.20)	38.01 (31.75–44.27)	113.42 (98.59–128.25)	138.62 (123.79–153.45)	153.90 (139.07–168.73)	63.22 (54.54–71.91)	169.54 (144.84–194.24)
	**Post**_**30min**_	44 (41–48)	26.73 (20.47–32.99)	35.35 (29.09–41.61)	39.28 (33.02–45.54)	110.85 (96.02–125.68)	131.07 (116.24–145.90)	**161.05**^**^^**^ **(146.22–175.88)**	60.71 (52.03–69.39)	129.92 (102.24–157.61)
**Young Adults**	**Pre**	**42**^**#**^ **(38–46)**	22.50 (16.06–28.94)	34.54 (28.10–40.99)	43.00 (36.56–49.44)	94.39 (79.13–109.66)	129.32 (114.04–144.61)	147.22 (131.92–162.52)	58.05 (48.83–67.27)	157.09 (135.86–178.31)
	**Post**_**5min**_	40 (36–44)	22.01 (15.56–28.45)	31.97 (25.53–38.41)	44.56 (38.12–51.00)	96.59 (81.34–111.86)	128.19 (112.93–143.45)	151.48 (136.22–166.74)	67.48^**^ **(58.56–76.41)**	156.22 (133.54–178.89)
	**Post**_**30min**_	40 (36–44)	21.99 (15.55–28.43)	32.82 (26.38–39.26)	46.63 (40.18–53.07)	**105.87**^**^^^**^ **(90.61–121.13)**	**136.23**^**^**^ **(120.97–151.49)**	**156.30**^**^^**^ **(141.03–171.57)**	68.88^**^ **(59.69–78.06)**	167.00 (144.62–189.39)

*iM1 ipsilateral primary motor cortex AMT* active motor threshold*, MEP* motor evoked potential, *M*_*MAX*_ maximum compound action potential, *SICI* short-interval intracortical inhibition, *ICF* intracortical facilitation. *(*p* < 0.01) indicates significant release of intracortical inhibition SICI; and ^^^ (*p* < 0.05), ^^^^(*p* < 0.04), ^^^^^(*p* < 0.001**)** indicate significant increase in cSP at 5- and 30-min post exercise from baseline. ^#^ (*p* < 0.05) denotes significant difference in AMT between the two groups at baseline

### Early and Late Phase MEPs

For changes in the early MEP, a significant main effect was observed for MSO (F_3, 385_ = 98.51, p < 0.001) and age group (F_1, 35_ = 8.43, p = 0.006), along with a significant MSO × age group interaction (F_3, 385_ = 8.27, p < 0.001), but not for time (F_2, 385_ = 2.30, p = 0.101). Young adults exhibited significantly higher early MEPs than older adults at 130%, 150%, and 170% AMT (MD = 0.49 to 0.97 mV, all p < 0.05, d = 0.69 to 0.83). However, no significant effect of exercise was seen in either group on the corticospinal tract in the iM1.

**Fig. 4:**
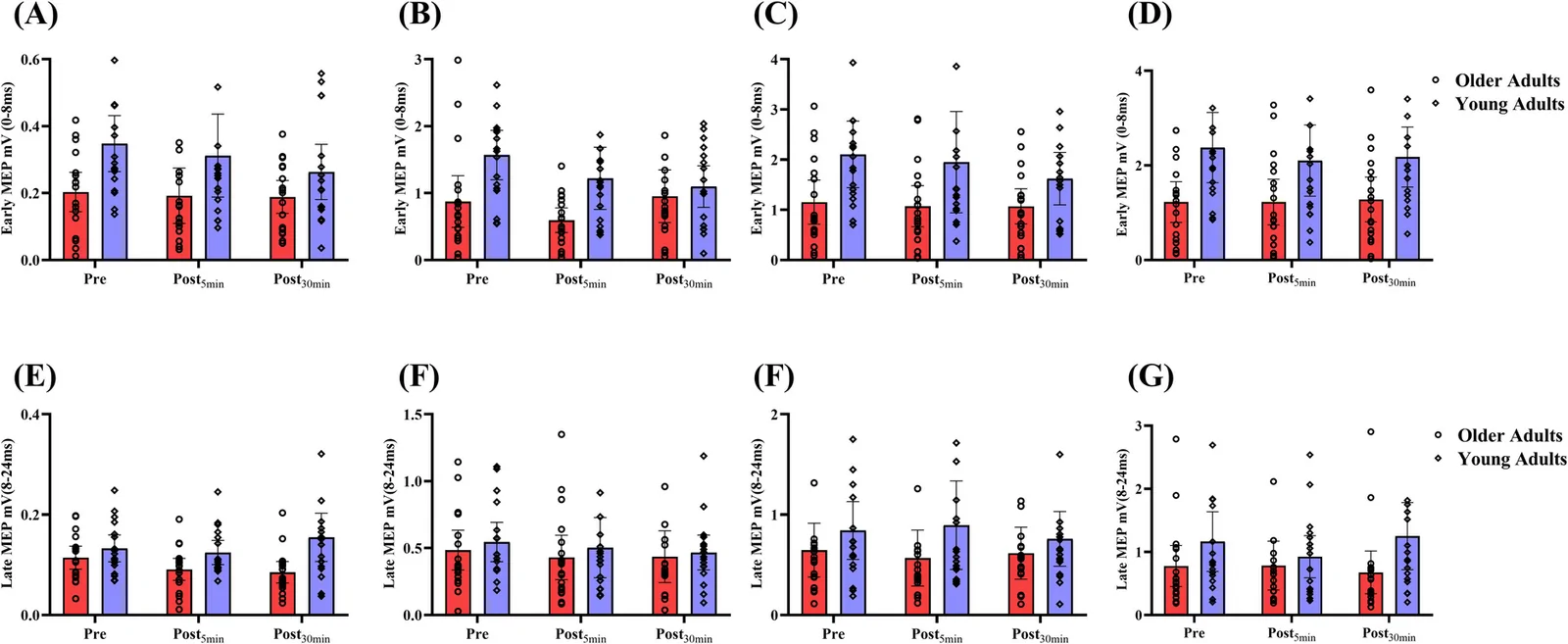
MEP amplitude (Mean ± 95%CI) during the early MEP phase at (**A**) 100% AMT, (**B**) 130% AMT, (**C**) 150% AMT and (D) 170% AMT, and during the late MEP phase at (**E**) 100% AMT, (**F**) 130% AMT, (**G**) 150% AMT and (**H**) 170% AMT in both older and younger adults. Data are presented for all time points before and after exercise

For the late component, a significant main effect was observed for MSO (F_3, 385_ = 84.47, p < 0.001), but not for time (F_2, 385_ = 0.57, p = 0.568) or age group (F_1, 35_ = 2.04, p = 0.162). However, a significant MSO × age group interaction (F_3, 385_ = 4.15, p = 0.007) was observed, with significantly higher late MEPs in young adults than older adults at 170% AMT (MD = 0.37, p = 0.009, 95% CI [0.094, 0.641], d = 0.47). No effect of exercise was seen in either group on the late MEPs (Fig. 4).

### Cortico-cortical measures (SICI & ICF)

**Fig. 5:**
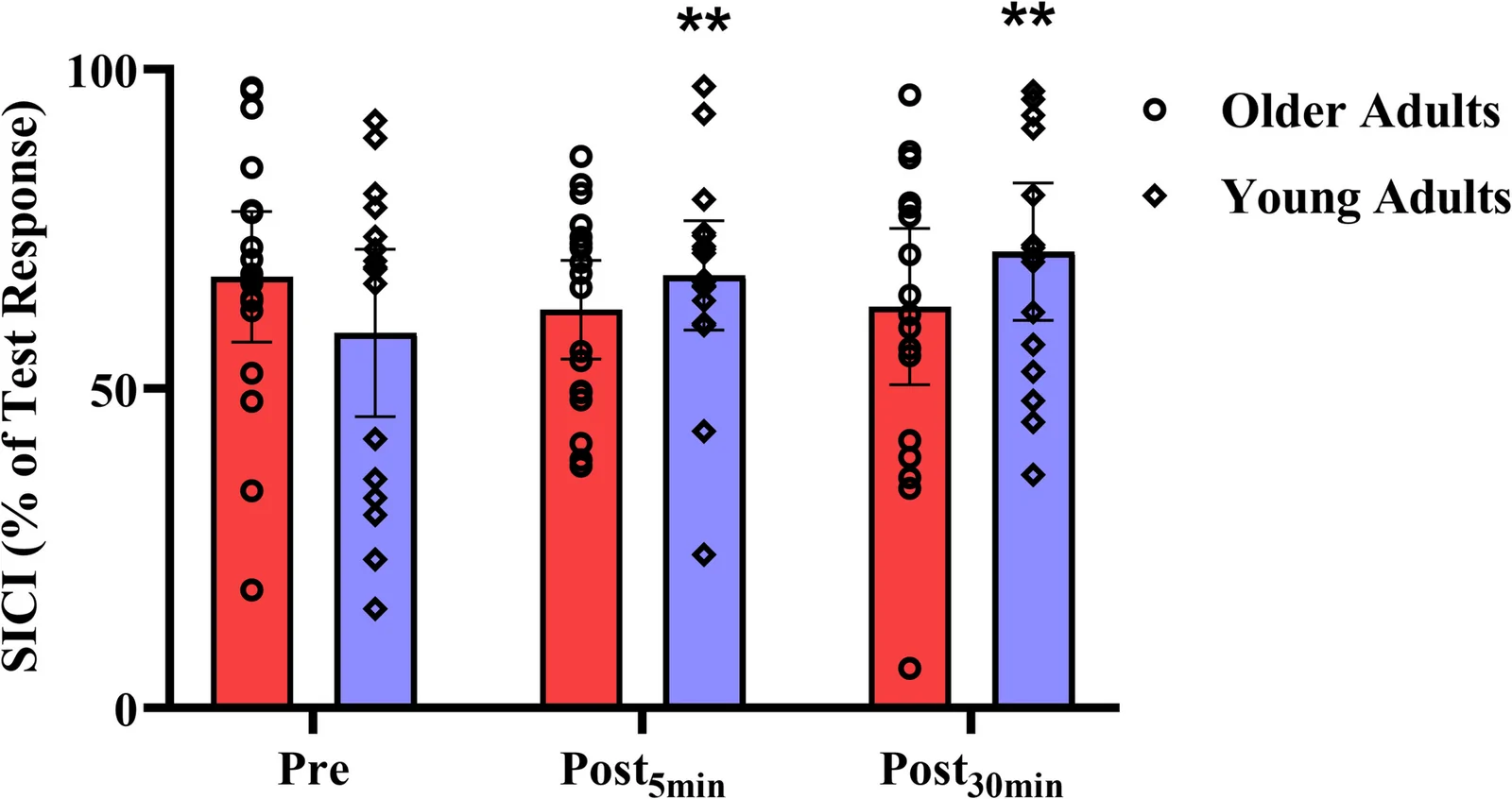
SICI (Mean ± 95%CI) of the untrained iM1 in both older and young adults. Significant release of SICI is denoted by **(*p* < 0.01) in young adults at 5- and 30-min post exercise

Analysis of SICI revealed no significant main effects for time (F_2, 369_ = 1.13, p = 0.325) or age group (F_1, 35_ = 0.79, p = 0.780). However, a significant time × age group interaction was observed (F_2, 369_ = 5.54, p = 0.004). Post hoc analysis indicated no significant changes from baseline in older adult’s post-exercise. In contrast, younger adults exhibited increased SICI from baseline at 5- and 30-min post-exercise (MD = 1.39 to 10.83, all p = 0.01, *d* = 0.24 to 0.56) (Fig. 5).

Examination of ICF showed no significant main effects for time (*F*_2, 283_ = 0.89, *p* = 0.41), or age group (*F*_1, 34_ = 1.52, *p* = 0.23) and no significant time × age group interaction (*F*_2,283_ = 1.82, *p* = 0.16) (Table 2).

### Cross activation and laterality index (LI)

For measures of cross activation, a significant fixed effect was observed for time (*F*_1, 30_ = 13.35, *p* < 0.001) while no significant effects were found for age group (*F*_1, 30_ = 0.53, *p* = 0.471) or the time × age group interaction (*F*_1, 30_ = 3.07, *p* = 0.090). However young adults exhibited greater cross activation (Pre: 27.39 ± 36.72 vs Post: 44.35 ± 50.84) than older adults (Pre: 43.32 ± 36.66 vs Post: 49.28 ± 40.28).

For the laterality Index (LI) for corticospinal excitability, significant fixed effects were observed for time (*F*_2, 280_ = 60.06, *p* < 0.001), MSO (*F*_2, 280_ = 72.89, *p* < 0.001) and age group (*F*_1, 35_ = 12.15, *p* < 0.001). However, the time × age group interaction was not significant (*F*_2, 280_ = 0.24, *p* = 0.78) suggesting that the temporal pattern of change did not differ between the two groups.

**Fig. 6:**
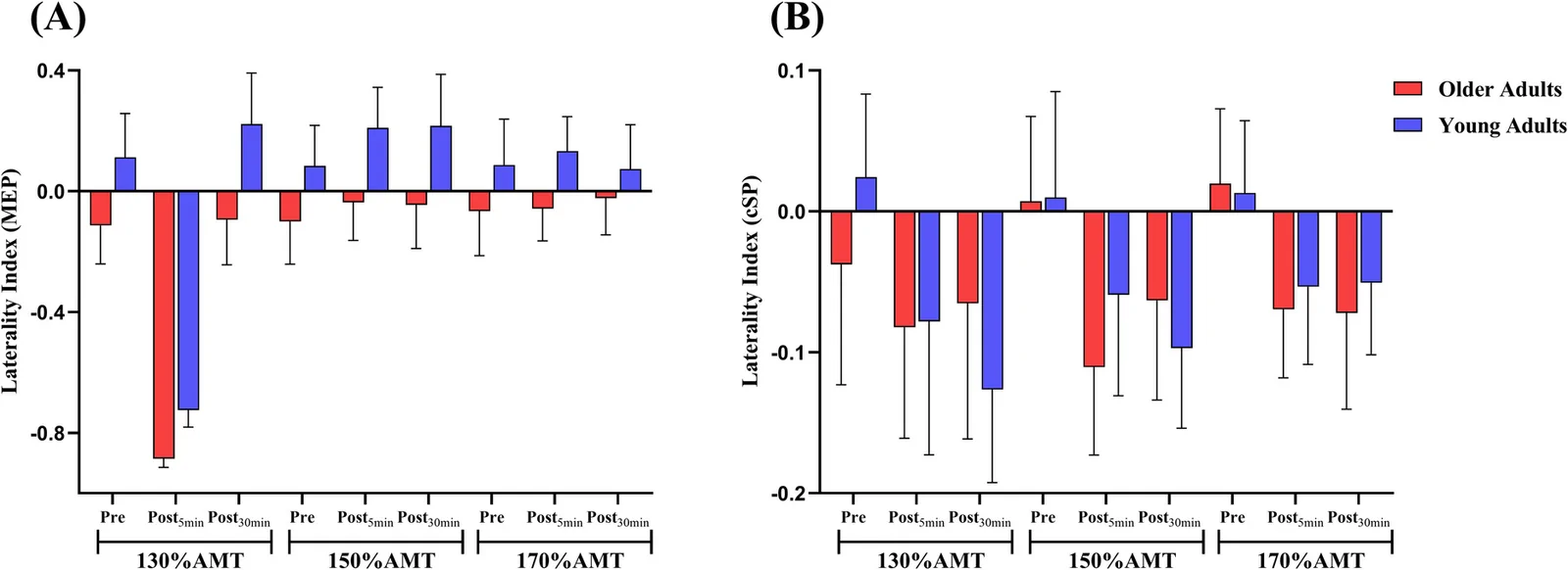
Laterality Index for (**A**) MEP and (**B**) cSP (Mean ± 95%CI) in both older and young adults across 130%, 150% and 170%AMT

For the LI of the cSP measure, a significant fixed effect was observed for time (*F*_2, 280_ = 33.43, *p* < 0.001), while no significant effects were found for MSO (*F*_2, 280_ = 2.40, *p* = 0.09), age group (*F*_1, 35_ = 0.03, *p* = 0.86) or the time × age group interaction (*F*_2, 280_ = 2.56, *p* = 0.08). These results indicated that LI for cSP significantly changed across time points following exercise but did not differ between age groups (Fig. 6, Table 3).

**Table 3:** Mean (95% CI) for laterality index prior to and following unilateral strength training in older and young adults

		**Older Adults**			**Young Adults**		
		**Pre**	**Post**_**5min**_	**Post**_**30min**_	**Pre**	**Post**_**5min**_	**Post**_**30min**_
Laterality Index (MEP)	130% AMT	−0.11 (−0.23–0.01)	−0.89 (−1.01–0.77)	−0.09 (−0.21–0.03)	0.11 (−0.01–0.24)	−0.72 (−0.85–0.60)	0.22 (0.10–0.34)
	150% AMT	−0.09 (−0.22–0.02)	−0.04 (−0.16–0.08)	−0.04 (−0.16–0.07)	0.08 (−0.04–0.21)	0.21 (0.09–0.33)	0.22 (0.09–0.34)
	170% AMT	−0.07 (−0.18–0.05)	−0.06 (−0.18–0.06)	−0.02 (−0.14–0.09)	0.09 (−0.04–0.21)	0.13 (0.01–0.26)	0.07 (−0.05–0.19)
Laterality Index (cSP)	130% AMT	−0.04 (−0.09–0.02)	−0.08 (−0.14–0.02)	−0.06 (−0.13–0.00)	0.02 (−0.04–0.09)	−0.08 (−0.14–0.02)	−0.13 (−0.19–0.06)
	150% AMT	0.01 (−0.06–0.07)	−0.11 (−0.017–0.05)	−0.06 (−0.13–0.00)	0.01 (−0.05–0.07)	−0.06 (−0.12–0.00)	−0.09 (−0.16–0.03)
	170% AMT	0.02 (−0.04–0.08)	−0.07 (−0.13–0.01)	−0.07 (−0.13–0.01)	0.01 (−0.05–0.08)	−0.05 (−0.12–0.01)	−0.05 (−0.11–0.01)

*MEP motor evoked potential; cSP: cortical silent period; AMT: active motor threshold*

### Central activation ratio (CAR)

Analysis of neural drive following unilateral strength training revealed no significant differences in both older (Pre 97.48 ± 4.02 vs Post_5min_ 97.83 ± 1.98 and Post_30min_ 98.36 ± 1.97) and young adults (Pre 98.93 ± 0.58 vs Post_5min_ 99.13 ± 0.59 and Post_30min_ 99.16 ± 0.46). There were no significant main effects for time (*F*_2, 65_ = 1.32, *p* = 0.27), age group (*F*_1, 33_ = 3.85, *p* = 0.06), or the time x age group interaction (*F*_2, 65_ = 0.52, *p* = 0.59) post-exercise.

### StartReact test

**Fig. 7:**
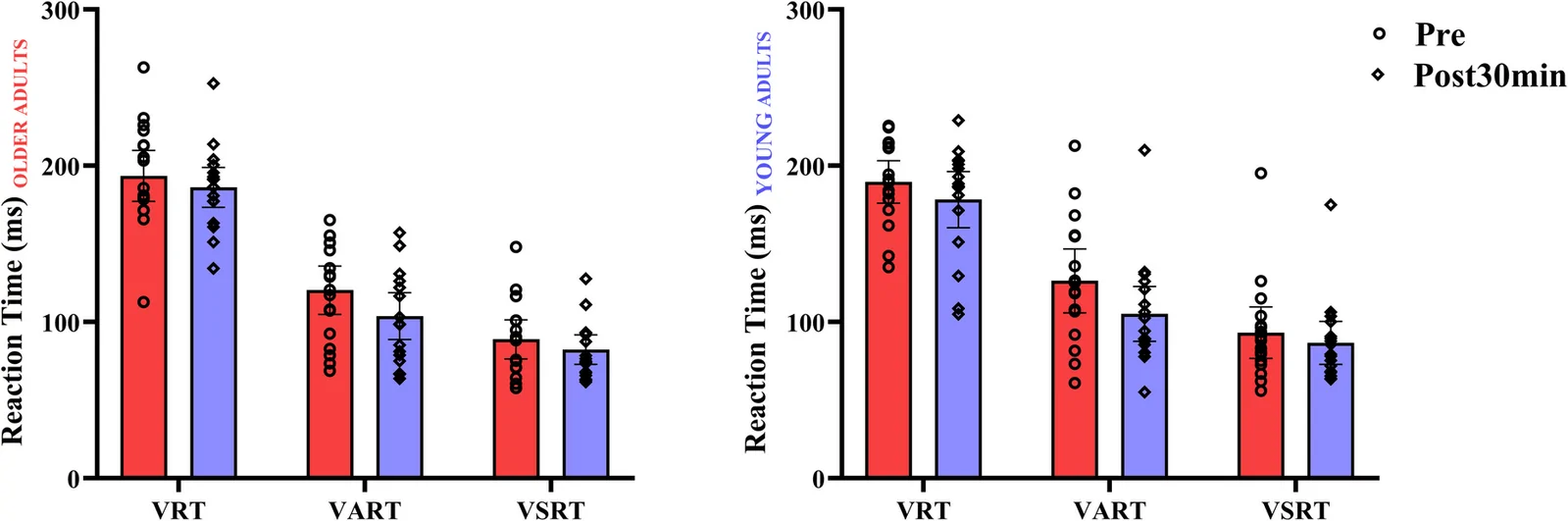
Reaction times (ms) (Mean ± 95%CI) for VRT (visual-only), VART (light + soft sound), and VSRT (light + startling sound) in (**A**) older and (**B**) younger adults pre- and post-exercise

The analysis of reaction time revealed significant main effects for time (*F*_1, 175_ = 15.93, *p* < 0.001) and condition (VRT, VART and VSRT; *F*_2, 175_ = 419.17, *p* < 0.001). Reaction time for the startling sound condition was significantly lower from the soft and no sound conditions (p < 0.001). While reaction time significantly changed from baseline (p < 0.001), it did not differ between age groups (*F*_1, 35_ = 0.01, *p* = 0.925) (Fig. 7, Table 4).

**Table 4:** Mean (95% CI) for the change in Reaction time, SREffect, RFD across different sound conditions in the untrained limb for older and young adults following a session of MPST

						**RFD (N.m/s)**					
		**Reaction Time (ms)**				**VRT**		**VART**		**VSRT**	
		**VRT**	**VART**	**VSRT**	**SR**_**Effect**_	**0–50 ms**	**50–100 ms**	**0–50 ms**	**50–100 ms**	**0–50 ms**	**50–100 ms**
Older Adults	Pre	193.53 (179.81–207.25)	120.39 (106.67–134.11)	88.93 (75.21–102.66)	31.46 (21.90–41.01)	485.39 (397.21–573-56)	217.92 (129.74–306.09)	482.92 (394.74–571.09)	212.16 (123.98–300.33)	532.05 (443.87–620.22)	192.86 (104.68–281.03)
	Post_30min_	186.22 (172.49–199.94)	103.79 (90–06-117.51)	82.37 (68.65–96.09)	21.42 (11.86–30.97)	446.18 (358.00–534.35)	232.57 (144.39–320.74)	485.28 (397.10–573.45)	231.19 (143.01–319.36)	520.67 (432.49–608.85)	222.68 (134.51–310.86)
Young Adults	Pre	189.65 (175.53–203.77)	126.36 (112.24–140.48)	93.19 (79.07–107.31)	33.17 (23.34–43.00)	763.33 (672.59–854.06)	290.04 (199.30–380.77)	765.55 (674.82–856.28)	320.63 (229.89–411.36)	912.70 (821.97–1003.44)	308.05 (217.32–398.78)
	Post_30min_	178.37 (164.25–192.49)	105.23 (91.11–119.35)	86.68 (72.56–100.79)	18.55 (8.72–28.39)	675.29 (584.56–766.03)	236.78 (146.05–327.52)	643.18^**^ (552.45–733.91)	265.52 (174.78–356.25)	791.02^**^ (700.29–881.75)	249.49 (158.76–340.23)

*SREffect* startreact effect*, RFD* rate of force development, *VRT* visual reaction time, *VART* visual-acoustic reaction time, *VSRT* visual startle reaction time

^**^ (p < 0.01) indicates significant decrease at 30 min post exercise from baseline in young adults

**Fig. 8:**
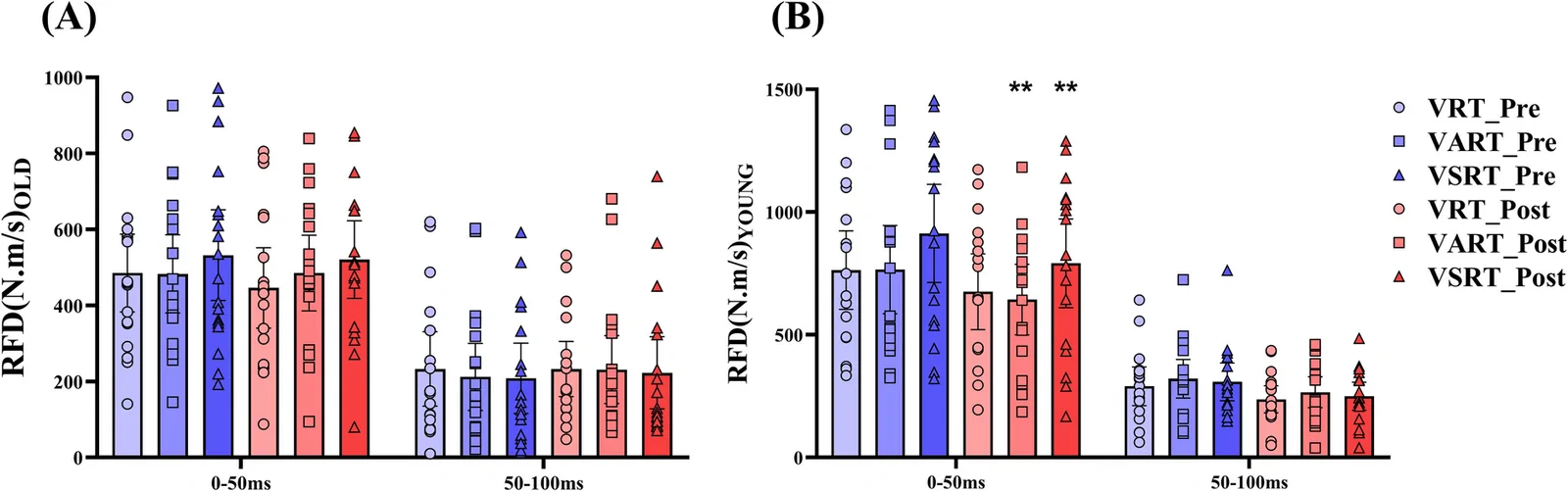
Force production (Mean ± 95%CI) of StartReact responses in older (**A**) and younger (**B**) adults. Rate of force development (RFD) is shown in (A-B), analysed over two-time windows (0–50 ms and 50–100 ms). Responses were compared between three StartReact cues: light only (VRT), light with a quiet sound (VART), and light with a startling sound (VSRT). **(*p* < 0.01) denotes significant decrease in RFD for VART and VSRT condition within 0–50 ms post exercise in young adults

For RFD significant main effect was found for time (*F*_1, 594_ = 8.60, *p* = 0.003), age group (*F*_1, 35_ = 5.01, *p* = 0.032) and time × age group interaction (*F*_1, 594_ = 13.40, *p* < 0.001). Older adults showed no significant changes in RFD across all sound conditions and time intervals. In contrast, young adults exhibited reduced RFD at Post_30min_ of exercise for the VART and VSRT conditions in the 0–50 ms interval (MD = −121.68 to −122.37, all *p* < 0.005, *d* = 0.84 to 0.86). Significant between-group differences in RFD were also observed at baseline and Post_30min_ of exercise for all sound conditions in the 0–50 ms interval (*p* < 0.05). Young adults exhibited greater RFD than the older adults (Fig. 8, Table 4).

For EMGrms data collected during StartReact, a significant time × age group interaction was found (*F*_1, 594_ = 53.24, *p* < 0.001) with significant fixed effect for time (*F*_1, 594_ = 15.85, *p* < 0.001 and age group (*F*_1, 35_ = 12.01, *p* = 0.001). While no significant changes were observed in older adults, whereas young adults showed a significant decrease in EMGrms for the VRT condition in the 50–100 ms intervals and for VART and VSRT condition in both the 0–50 ms and 50–100 ms intervals (MD = −0.017 to −0.035, all *p* < 0.05, *d* = 0.36 to 0.52). For between-group comparisons, a significant difference was observed between older and young adults at baseline for all sound conditions in both the 0–50 ms and 50–100 ms intervals (*p* < 0.005). At Post_30min_ of exercise, significant differences were seen between the groups for all sound conditions in the 0–50 ms interval only (*p* < 0.05).

## Discussion

This study examined acute neurophysiological responses in the untrained hemisphere following a single session of metronome-paced unilateral strength training, with a specific focus on corticospinal, corticoreticular, and reticulospinal pathway dynamics. Comparisons between younger and older adults revealed distinct age-related patterns of neural responsiveness. Corticospinal excitability, as measured by MEPs, did not significantly change in either cohort post-exercise. However, cSP duration, reflecting GABA_B_-mediated intracortical inhibition, increased across all stimulation intensities in younger adults, while older adults demonstrated a significant prolongation only at the highest intensity (170% AMT) within the iM1. Short-interval intracortical inhibition (SICI), indicative of GABA_A_-ergic tone, was released at 30 min post-exercise in younger adults, with no corresponding modulation observed in older participants. Despite stable early- and late-phase MEP amplitudes across time points, younger adults consistently exhibited greater early-phase MEP amplitudes across all TMS intensities, suggesting increased baseline corticospinal excitability. No significant post-exercise changes were detected in reticulospinal excitability in either group, as assessed via the StartReact paradigm. While RFD declined acutely in younger adults, it remained significantly greater than that of older adults in response to both VART and VSRT cues. Voluntary activation, measured via central activation ratio (CAR), remained unchanged in both groups. Collectively, these results highlight age-dependent differences in the ability for the induction of neuroplasticity, particularly within inhibitory and corticospinal circuits, and underscore potential limitations in subcortical plasticity with ageing. These findings provide mechanistic insight into the differential expression of cross-education across the lifespan.

### Evidence for corticoreticular & reticulospinal contributions in the untrained limb following UST

This study is the first to examine acute changes in the corticoreticular and reticulospinal pathways in the untrained limb following a single session of metronome-paced unilateral strength training. No significant post-exercise changes were observed in early- or late-phase MEPs in either young or older adults, suggesting limited acute recruitment of these pathways in the untrained hemisphere. However, young adults exhibited greater early-phase MEP amplitudes across all TMS intensities, and greater late-phase MEPs at 170% of AMT, compared to older adults. These between-group differences point to age-related differences in overall responsiveness of descending motor pathways.

Early-phase MEPs are commonly attributed to corticospinal tract activation, while late-phase MEPs are thought to reflect polysynaptic cortico-brainstem circuits, including the cortico-reticulospinal tract [9, 36, 42]. The absence of post-exercise modulation in either phase in both groups indicates that neither the corticospinal tract nor the cortico-reticulospinal tract was acutely facilitated in the untrained hemisphere. This finding contrasts with the hypothesis that older adults might show increased reliance on the cortico-reticulospinal tract to compensate for age-related corticospinal tract decline [120]. Despite well-documented corticospinal tract degeneration in ageing [85, 101], our results did not show evidence of compensatory activation of the cortico-reticulospinal tract. One potential explanation is reduced interhemispheric plasticity in older adults, possibly related to age-associated structural decline of the corpus callosum [75], which may limit the effectiveness of neural crossover after unilateral training.

In young adults, the presence of greater early-phase MEP amplitudes across TMS intensities and a longer cortical silent period post-exercise may reflect a complex interaction between excitatory and inhibitory processes. However, the lack of significant change in MEP amplitude post-exercise suggests no acute enhancement of corticospinal tract output. Furthermore, the absence of changes in late-phase MEPs indicates that alternative descending pathways, such as the cortico-reticulospinal tract, were not acutely recruited. These findings highlight distinct age-related differences in motor pathway responsiveness, with greater MEP amplitudes observed in young adults across stimulation intensities. However, the lack of post-exercise changes in either group suggests that acute corticospinal and cortico-reticulospinal plasticity may be limited following a single session of unilateral strength training.

To further assess the contribution of the reticulospinal tract to neural adaptations underlying CE, we employed the StartReact paradigm to evaluate reticulospinal tract excitability in the untrained limb [26]. No significant post-exercise changes were observed in either group for VSRT reaction time. Older adults showed a non-significant 19% reduction in VSRT, and both groups exhibited moderate, non-significant decreases in SR_Effect_, with a 32% reduction in older adults (d = 0.5). The StartReact response reflects subcortical facilitation via the pontomedullary reticular formation, enhancing α-motoneuron recruitment [14, 15, 114]. Despite this mechanism's relevance to rapid motor output, our findings suggest that a single session of metronome-paced unilateral strength training does not acutely modulate reticulospinal tract excitability. This likely reflects an insufficient training stimulus in terms of intensity or volume. While previous studies link greater strength with enhanced reticulospinal tract excitability [1, 110] and chronic training with reticulospinal tract facilitation [53], our results suggest that such adaptations require more extended training. The observed SR_Effect_ reductions may indicate transient neural adjustments, such as motor learning or mild fatigue, rather than reticulospinal tract plasticity. Additionally, age-related degeneration of brainstem structures [8, 74] may reduce reticulospinal tract responsiveness in older adults, potentially contributing to the attenuated StartReact responses.

Beyond reaction time, startling stimuli enhance RFD and muscle activity [2, 41]. Increased RFD reflects faster motor unit recruitment, higher discharge rates, and improved synchronization [34, 38], largely influenced by subcortical pathways such as the reticulospinal tract [109]. In our study, RFD was greater in the VSRT condition compared to VART and VRT, supporting the role of the reticular formation in rapid force generation within the 0–50 ms window, as cortical involvement is unlikely in this timeframe [14, 87]. Post-exercise, younger adults exhibited a significant reduction in RFD in the untrained limb, while older adults showed no change. This may reflect acute modulation of subcortical output in younger adults, potentially influenced by fatigue in the trained limb. The lack of response in older adults may reflect reduced adaptability of these pathways, aligning with reported age-related declines in reticulospinal function [81]. Future studies with greater training volume or duration may help determine whether prolonged activation elicits measurable changes in reticulospinal excitability.

Muscle excitability (EMGrms) during the StartReact task was assessed to evaluate motor output in response to startling stimuli, reflecting subcortical drive to the motoneuron pool, including reticulospinal contributions [103, 110, 115]. Both groups showed increased EMGrms in the VSRT condition compared to VRT and VART, indicating enhanced motor unit activity under startling conditions. Post-exercise, younger adults exhibited reduced EMGrms in both early and late windows, while older adults showed no significant change. Between-group differences were present at baseline and post-exercise, with greater variability in younger adults, suggesting age-related differences in subcortically mediated motor responses. These acute changes may not fully reflect underlying neuroplasticity, which may require repeated training to elicit sustained adaptations.

Our results reveal age-related differences in acute neural responses to unilateral strength training. Although no post-exercise changes were detected in either group, younger adults showed consistently greater corticospinal and subcortical output, indicating preserved neural responsiveness. These findings reflect short-term neural activity rather than CE, which typically requires repeated training [4, 16]. Future studies should assess whether longer-term training induces reticulospinal plasticity and contributes to strength transfer.

### Ipsilateral corticospinal excitability and cortical silent period

Corticospinal excitability in the untrained hemisphere (iM1) showed no significant changes post-exercise in either group across all motor threshold levels. Additionally, no between-group differences were observed in corticospinal excitability changes from pre- to post-exercise. Literature on acute motor training, particularly ballistic tasks, reports both increases [18, 64, 96] and decreases [7, 39] in MEP amplitudes, with some studies showing no changes [88]. These discrepancies suggest that corticospinal excitability responses are highly task-dependent [70, 93, 95]. The absence of studies on corticospinal excitability changes following acute metronome-paced strength training in older adults makes it difficult to interpret the lack of change in this group. Our results in young adults align with other studies on acute strength training, which also report no changes in corticospinal excitability [54, 56, 65], although some have observed increases [13, 80]. A systematic review concluded that there is no clear correlation between MEP amplitude changes and strength gains, suggesting that neural mechanisms beyond the corticospinal tract may contribute to CE [25].

Beyond corticospinal excitability, cross activation, which drives neural adaptations in circuits projecting to the untrained limb (iM1), was not significantly altered in either group post-exercise. In older adults, this suggests limited acute neural adaptation, possibly due to age-related plasticity constraints [108]. In contrast, young adults showed a 62% (d = 0.4) increase in cross activation, consistent with prior studies [46, 59, 60], reflecting greater neural adaptability, likely facilitated by better interhemispheric communication. The lack of evidence on cross activation in older adults highlights a significant research gap. Additionally, lower fatigue in older adults, as shown by MVF changes in the trained limb, and age-related declines in interhemispheric communication and neural conduction may limit cross activation [119].

For corticospinal inhibition, we observed a significant increase in iM1 inhibition post-exercise in young adults across all TMS intensities, while older adults showed increased inhibition only at 170% AMT. The silent period, primarily mediated by GABA-_B_ inhibition, plays an important role in regulating cortical excitability [117] and is associated with CE mechanisms [45]. While previous studies generally report a reduction in intracortical inhibition following strength training in young adults [27, 55, 71], our findings of increased inhibition are novel. This suggests that both young and older adults experience increased inhibition in the untrained hemisphere in an age-dependent manner after a single session of unilateral strength training. In young adults, the increase in inhibition may be linked to enhanced interhemispheric communication, allowing for more precise modulation of neural activity post-exercise. This increased inhibition could act as a protective mechanism, preventing excessive excitation and ensuring balanced neural activity, thus supporting better motor control and performance [98]. However, in older adults, the increased silent period duration may reflect compensatory transcallosal inhibition (TCI) from the trained hemisphere to the untrained iM1 [75], potentially influenced by high-intensity exercise [19]. Age-related declines in synaptic plasticity and neural processing speed may limit the ability to fully adapt to strength training, potentially affecting the modulation of intracortical inhibition [119]. Further research is needed to explore these age-related changes in the context of strength training.

### Changes ipsilateral intracortical mechanism and hemispheric lateralization

SICI, primarily mediated by GABA-_A_ receptors, plays a critical role in modulating motor cortex excitability in the untrained hemisphere (iM1) [121]. Reductions in SICI, reflected by higher SICI ratios (i.e., less conditioned MEP suppression relative to unconditioned MEP) indicate intracortical disinhibition and has been consistently reported in young adults following unilateral strength training [67, 80, 82, 92]. This disinhibition is believed to facilitate CE by enhancing cortical excitability and interhemispheric communication.

In the present study, younger adults demonstrated a significant post-exercise reduction in SICI, consistent with previous reports. In contrast, older adults exhibited no significant change, suggesting diminished intracortical inhibitory plasticity with age. These findings are partially consistent with earlier studies involving acute motor training in older adults, some of which also reported no SICI modulation [21, 97], while others observed task-dependent increases [64, 88]. Such discrepancies may be attributed to differences in task type, intensity, and methodological approaches [92, 122]. The absence of SICI modulation in older adults may reflect structural and functional age-related changes, including corpus callosum atrophy [75] and elevated transcallosal inhibition [43], which may constrain the capacity for short-term intracortical plasticity. These results highlight a potential mechanistic limitation in the older motor system's responsiveness to unilateral strength training.

We observed no significant post-exercise changes in ICF in either group. Currently, there is limited direct evidence on ICF’s role in acute neural responses [24, 45, 82]. However, some studies suggest that motor practice can modulate intracortical excitability, influencing both inhibitory and excitatory circuits [20]. Since there is no clear evidence that ICF changes translate into functional improvements in the untrained limb, further research is needed to clarify its role in acute neural responses.

Hemispheric lateralization may influence CE by modulating neural adaptations in the untrained limb. This refers to the functional specialization of the brain's hemispheres [11, 35]. Unilateral training can induce changes in the trained hemisphere that affect the untrained limb through interhemispheric communication, as suggested by the bilateral access hypothesis [98]. Our findings revealed distinct interhemispheric asymmetries in corticospinal excitability across both age groups. However, the temporal pattern of change did not differ between age groups, suggesting similar shifts in corticospinal excitability following exercise. Our use of a laterality index to track post-exercise shifts in interhemispheric excitability and inhibition provides a novel approach to quantifying bilateral cortical reorganization after unilateral motor training. Notably, the post-exercise lateralization of the silent period (cSP) was more pronounced than that of corticospinal excitability, indicating that changes in transcallosal inhibition may be a more sensitive marker of interhemispheric reorganization. Significant changes in silent period were noted over time, yet no differences were found between age groups. This supports the idea that both young and older adults demonstrate similar lateralization patterns post-exercise, despite potential differences in neural mechanisms such as transcallosal inhibition [111] or neural plasticity [105]. These results underscore the role of hemispheric lateralization in CE and highlight the need for further investigation into age-related differences in interhemispheric communication. While both age groups exhibited shifts in corticospinal excitability, the underlying mechanisms may differ due to age-related neural changes [11]. Further research is needed to explore these mechanisms and their implications for neuroplastic adaptations and CE across the lifespan.

### Effect of unilateral strength training on overall voluntary drive

Functional improvements in the untrained limb through CE are known to originate from neural mechanisms, observable after a single session of strength training (Leung et al., 2015). To assess neural drive to the motoneuron pool of the untrained limb, we measured the Central Activation Ratio (CAR) [47, 73]. No significant changes in CAR were observed in either group. Although no immediate changes were detected, chronic studies suggest that neural adaptations in contralateral pathways may require multiple training sessions [77, 107]. The absence of significant CAR changes in both groups may indicate that CE mechanisms take longer to fully manifest, particularly in older adults.

## Limitations and future direction

While the findings of this study are new and noteworthy, there are several limitations to consider. First, although all participants self-reported their sedentary status, the outcomes may have differed had individuals with higher levels of physical activity been included. Second, assessing the integrity of the cortico-reticular pathway through late-phase MEPs is a novel approach that has not yet been fully validated. Using iMEPs measurements could have provided a more robust assessment. Third, the number of outcome measures and the repeated testing may have contributed to participant fatigue, potentially influencing recovery. Fourth, we did not perform a formal responder vs. non-responder analysis, which may have limited our ability to fully capture inter-individual variability in neural responsiveness, particularly among older adults. While group-level effects were the primary focus of this study, future work with larger samples is needed to explore variability in training-induced plasticity at the individual level. Finally, there was an uneven distribution of males and females across the groups, which may introduce gender-related bias. This imbalance likely arose from participant availability or recruitment patterns, and while it could affect the generalizability of the findings, particularly if sex-based differences influence neural or recovery responses, follow-up analysis using linear regression indicated that gender did not significantly influence the outcome measures, suggesting that the imbalance did not affect the overall results.

In addition, although this study focused on the acute neurophysiological responses following a single session of unilateral strength training, it is important to acknowledge that CE, as a functional adaptation, generally requires multiple training sessions to fully develop. Our findings offer preliminary insight into early-phase neurophysiological changes that may contribute to CE mechanisms, particularly in older adults where such responses remain underexplored. Future research employing prolonged training interventions is warranted to delineate the temporal dynamics and maximal adaptive capacity of both cortical and subcortical motor pathways in this population.

## Conclusion

This study demonstrates distinct, age-dependent neurophysiological adaptations in the untrained hemisphere following a single session of unilateral strength training. Although neither age group showed significant changes in corticospinal excitability, notable age-related differences emerged in intracortical inhibition mechanisms. Young adults exhibited greater cortical silent period inhibition across all TMS intensities, suggesting a more robust inhibitory response. In contrast, older adults showed similar adaptations only at higher stimulation intensities, indicating reduced sensitivity in intracortical inhibition. Similarly, for short-interval intracortical inhibition (SICI), which reflects GABA_A_-mediated inhibitory circuits, young adults demonstrated a more flexible and responsive inhibitory mechanism supporting the CE effect, whereas older adults exhibited a more constrained adaptation. No significant age-related differences were observed in early or late MEP modulation post-exercise, indicating limited acute changes in corticospinal and cortireticulopinal dynamics. Additionally, neither group showed significant changes in reaction time to startling stimuli, suggesting minimal engagement of the reticulospinal system in the untrained limb following a single session of unilateral training. These findings highlight the complexity of acute neural plasticity in response to unilateral exercise and emphasize the importance of considering age-related differences in descending motor pathway responsiveness. A deeper understanding of these mechanisms may inform the development of more effective, pathway-specific rehabilitation strategies tailored to the neurophysiological characteristics of different age groups.

## Supplementary Information

Below is the link to the electronic supplementary material.

Supplementary file1 (DOCX 804 KB)

Supplementary file2 (DOCX 22 KB)
