# Supplementary material for: Novel evidence of age-related cortical and subcortical constraints in cross-education
Source: GeroScience. 2025 Oct 25;48(4):5485–508. doi: 10.1007/s11357-025-01886-1 (PMC13574755; doi:10.1007/s11357-025-01886-1)
Supplement: Supplementary file 1 [file 11357_2025_1886_MOESM1_ESM.docx]

**Supplementary Figure 1:**

Five overlay sweeps of single-pulse MEPs at 130%, 150% and 170%AMT from a single participant in both older and young group shown at baseline and post-training time points.

**Supplementary Figure 2:**

Rectified EMG data from a single participant in both older and younger groups, shown at baseline and post-exercise time points. TMS application is marked by an arrow in each panel. The x-axis shows time (ms); the y-axis shows EMG amplitude (mV), indicated by the thick vertical line. Time windows are labelled as early (E), reflecting corticospinal input, and late (L), representing corticoreticular or other corticofugal contributions.
