# Supplementary material for: Novel evidence of age-related cortical and subcortical constraints in cross-education
Source: GeroScience. 2025 Oct 25;48(4):5485–508. doi: 10.1007/s11357-025-01886-1 (PMC13574755; doi:10.1007/s11357-025-01886-1)
Supplement: Supplementary file 2 [file 11357_2025_1886_MOESM2_ESM.docx]

**Supplementary Table 1:** Participant characteristics for older and young adults stratified by sex. Values are mean (95%CI).

|  | **OLDER ADULTS** | | **YOUNG ADULTS** | |
| --- | --- | --- | --- | --- |
|  | **MALE** | **FEMALE** | **MALE** | **FEMALE** |
| **Number** | 6 | 12 | 7 | 10 |
| **Age (yrs)** | 68  (61-75) | 67  (65-70) | 28  (23-32) | 26  (20-32) |
| **Height (cm)** | 171.50  (160.76-182.24) | 164.75  (159.87-169.63) | 175.70  (168.62-182.78) | 162.14  (159.29-164.99) |
| **Weight (kg)** | 81.43  (63.53-99.33) | 72.20  (64.19-80.21) | 75.50  (68.03-82.97) | 59.43  (52.15-66.71) |
| **1RM (kg)** | 12  (9-15) | 8  (7-9) | 14  (12-16) | 6  (5-7) |

|  | | | **AMT** | **Corticospinal Excitability MEP (%M_MAX_)** | | | **Cortical Silent Period cSP (ms)** | | | **SICI** | **ICF** | **CAR** | **MVF** |
| --- | --- | --- | --- | --- | --- | --- | --- | --- | --- | --- | --- | --- | --- |
|  |  |  |  | **130%AMT** | **150%AMT** | **170%AMT** | **130%AMT** | **150%AMT** | **170%AMT** |  |  |  |  |
| **OLDER**  **ADULTS** | **MALE** | **Pre** | 45  (39-51) | 20.94  (10.15-31.74) | 36.64  (25.84-47.43) | 40.82  (30.02-51.61) | 118.82  (93.45-144.20) | 144.81  (119.43-170.18) | 161.38  (135.99-186.75) | 57.87  (43.63-72.11) | 159.95  (123.56-196.34) | 96.9  (95.2-98.5) | 115.40  (98.07-132.73) |
|  |  | **Post_5min_** | 44  (38-49) | 24.62  (13.83-35.41) | 32.14  (21.35-42.94) | 37.89  (27.09-48.68) | 117.03  (91.66-142.41) | 136.82  (111.44-162.19) | 145.60  (120.22-170.98) | 60.49  (46.43-74.54) | 176.31  (134.12-218.50) | 98.3  (96.7-100.0) | 117.71  (100.38-135.03) |
|  |  | **Post_30min_** | 43  (37-49) | 22.89  (12.09-33.68) | 30.63  (19.83-41.42) | 34.51  (23.72-45.30) | 118.47  (93.09-143.85) | 138.43  (113.05-163.80) | 166.88  (141.49-192.25) | 60.42  (46.17-74.66) | 121.92  (78.17-165.67) | 99.2  (97.5-100.1) | 116.70  (99.38-134.03) |
|  | **FEMALE** | **Pre** | 48  (43-53) | 28.51  (20.88-36.14) | 35.73  (28.9-43.36) | 39.61  (31.98-47.24) | 112.52  (94.57-130.46) | 128.74  (110.79-146.68) | 147.45  (129.50-165.39) | 69.74  (59.56-79.92) | 134.97  (109.24-160.69) | 97.8  (96.6-99.0) | 59.87  (47.62-72.12) |
|  |  | **Post_5min_** | 46  (42-51) | 21.70  (14.07-29.33) | 30.34  (22.71-37.97) | 38.08  (30.44-45.71) | 111.61  (93.67-129.56) | 139.52  (121.57-157.46) | 158.05  (140.10-175.99) | 64.51  (54.34-74.69) | 162.54  (136.76-188.33) | 97.6  (96.4-98.8) | 60.62  (48.37-72.87) |
|  |  | **Post_30min_** | 45  (40-50) | 28.65  (21.02-36.28) | 37.72  (30.08-45.35) | 41.67  (34.04-49.29) | 107.04  (89.09-124.99) | 127.39  (109.45-145.34) | 158.14  (140.19-176.08) | 60.78  (50.69-70.87) | 139.67  (110.03-169.32) | 98.0  (96.9-99.2) | 62.48  (50.23-74.74) |
| **YOUNG**  **ADULTS** | **MALE** | **Pre** | 42  (37-46) | 17.58  (9.22-25.94) | 33.43  (25.07-41.78) | 42.66  (34.30-51.02) | 96.04  (76.38-115.69) | 129.19  (109.54-148.85) | 141.96  (122.30-161.62) | 49.61  (38.03-61.19) | 163.46  (136.28-190.63) | 98.9  (97.6-100.3) | 119.08  (105.66-132.50) |
|  |  | **Post_5min_** | 39  (34-44) | 19.66  (11.30-28.02) | 30.53  (22.17-38.89) | 49.05  (40.69-57.41) | 95.08  (75.42-114.74) | 118.95  (99.29-138.61) | 142.94  (123.28-162.59) | 62.02  (50.92-73.12) | 169.81  (141.21-198.42) | 99.2  (97.9-100.6) | 112.82  (99.40-126.24) |
|  |  | **Post_30min_** | 39  (34-45) | 22.03  (13.67-30.39) | 31.38  (23.02-39.74) | 49.18  (40.82-57.54) | 102.89  (83.24-122.56) | 130.69  (111.04-150.36) | 146.67  (126.99-166.34) | 62.86  (51.74-73.99) | 182.14  (150.28-214.01) | 99.1  (97.7-100.4) | 106.86  (93.44-120.29) |
|  | **FEMALE** | **Pre** | 43  (34-52) | 29.54  (19.55-39.53) | 36.14  (26.15-46.13) | 43.49  (33.49-53.48) | 91.77  (68.25-115.29) | 128.87  (105.28-152.46) | 154.84  (131.18-178.51) | 69.42  (55.96-82.88) | 150.69  (118.04-183.35) | 99.0  (97.3-100.6) | 59.14  (43.10-75.18) |
|  |  | **Post_5min_** | 42  (33-50) | 25.36  (15.37-35.35) | 34.03  (24.04-44.02) | 38.15  (28.16-48.14) | 98.77  (75.27-122.26) | 141.39  (117.89-164.88) | 163.68  (140.18-187.17) | 75.29  (62.19-88.39) | 142.64  (107.41-177.87) | 99.0  (97.3-100.7) | 56.17  (40.13-72.21) |
|  |  | **Post_30min_** | 41  (34-49) | 21.93  (11.94-31.92) | 34.88  (24.89-44.87) | 42.97  (32.98-52.96) | 110.11  (86.62-133.61) | 144.14  (120.65-167.64) | 169.91  (146.42-193.41) | 77.55  (63.37-91.74) | 151.85  (120.36-183.33) | 99.3  (97.6-100.1) | 55..73  (39.69-71.77) |

**Supplementary Table 2:** Neurophysiological measures of corticospinal excitability (MEP amplitude at 130, 150, and 170% AMT), cortical silent period (cSP), short-interval intracortical inhibition (SICI), intracortical facilitation (ICF), central activation ratio (CAR), and maximal voluntary force (MVF) in older and young adults, stratified by sex. Data are presented as model-derived means (95% CI) from linear mixed models at baseline (Pre), 5 min post-training (Post5min), and 30 min post-training (Post30min).

**Supplementary Table 3:** Behavioural outcomes including reaction time (visual reaction time, VRT; visual–auditory reaction time, VART; visual–startle reaction time, VSRT) and rate of force development (RFD, 0–50 ms) in older and young adults, stratified by sex. Data are presented as model-derived means (95%CI) from linear mixed models at baseline (Pre) and 30 min post-training (Post30min).

|  | | | **Reaction Time (ms)** | | | **RFD (N/s) (0-50ms)** | | |
| --- | --- | --- | --- | --- | --- | --- | --- | --- |
|  |  |  | **VRT** | **VART** | **VSRT** | **VRT** | **VART** | **VSRT** |
| **Older**  **Adults** | **Male** | **Pre** | 176.28  (153.61-198.96) | 133.97  (11.29-156.65) | 110.18  (87.51-132.86) | 634.49  (522.69-746.31) | 619.54  (507.73-731.35) | 672.123  (560.31-783.93) |
|  |  | **Post_30min_** | 187.66  (164.98-210.33) | 130.25  (107.57-152.92) | 91.52  (68.85-114.20) | 594.95  (483.14-706.76) | 630.14  (518.33-741.95) | 652.34  (540.53-764.15) |
|  | **Female** | **Pre** | 202.15  (186.12-218.19) | 113.60  (97.57-129.64) | 78.31  (62.28-94.34) | 410.83  (331.77-489.89) | 414.61  (335.55-493.67) | 462.01  (382.95-541.07) |
|  |  | **Post_30min_** | 185.50  (169.47-201.53) | 90.56  (74.52-106.59) | 77.79  (61.76-93.83) | 371.79  (292.73-450.85) | 412.84  (333.78-491.90) | 454.83  (375.77-533.89) |
| **Young Adults** | **Male** | **Pre** | 190.99  (173.42-208.55) | 122.08  (104.51-139.64) | 89.95  (72.38-107.51) | 972.84  (886.23-1059.45) | 986.85  (900.24-1073.45) | 1197.45  (1110.84-1284.06) |
|  |  | **Post_30min_** | 177.56  (159.99-195.12) | 101.94  (84.37-119.49) | 85.81  (68.25-103.38) | 860.52  (773.91-947.13) | 827.58  (740.98-914.19) | 1030.14  (943.53-1116.75) |
|  | **Female** | **Pre** | 187.75  (166.75-208.74) | 132.49  (111.49-153.48) | 97.83  (76.83-118.82) | 464.03  (360.52-567.55) | 449.41  (345.89-552.92) | 505.92  (402.40-609.44) |
|  |  | **Post_30min_** | 179.54  (158.55-200.53) | 109.94  (88.94-130.93) | 87.91  (66.92-108.90) | 410.69  (307.18-514.21) | 379.75  (276.23-483.26) | 449.42  (345.90-552.93) |
